# Identified senescence endotypes in aged cartilage are reflected in the blood metabolome

**DOI:** 10.1007/s11357-023-01001-2

**Published:** 2023-11-14

**Authors:** Ilja Boone, Margo Tuerlings, Rodrigo Coutinho de Almeida, Johannes Lehmann, Yolande Ramos, Rob Nelissen, Eline Slagboom, Peter de Keizer, Ingrid Meulenbelt

**Affiliations:** 1 Section of Molecular Epidemiology, Department of Biomedical Data Sciences, Leiden University Medical Center, PO Box 9600, Post-zone S-05-P, 2300 RC Leiden, The Netherlands; 2 Center for Molecular Medicine, Division of Laboratories, Pharmacy and Biomedical Genetics, University Medical Center Utrecht, Utrecht, The Netherlands; 3 Department of Orthopaedics, Leiden University Medical Center, Leiden, The Netherlands; 4 Max Planck Institute for Biology of Aging, Cologne, Germany; 5 Cleara Biotech B.V., Utrecht, The Netherlands

**Keywords:** Osteoarthritis, Senescence, Blood-biomarkers, Endotypes, Metabolic blood profiles

## Abstract

Heterogeneous accumulation of senescent cells expressing the senescence-associated secretory phenotype (SASP) affects tissue homeostasis which leads to diseases, such as osteoarthritis (OA). In this study, we set out to characterize heterogeneity of cellular senescence within aged articular cartilage and explored the presence of corresponding metabolic profiles in blood that could function as representative biomarkers. Hereto, we set out to perform cluster analyses, using a gene-set of 131 senescence genes (*N* = 57) in a previously established RNA sequencing dataset of aged articular cartilage and a generated metabolic dataset in overlapping blood samples. Using unsupervised hierarchical clustering and pathway analysis, we identified two robust cellular senescent endotypes. Endotype-1 was enriched for cell proliferating pathways, expressing forkhead box protein O4 (*FOXO4)*, RB transcriptional corepressor like 2 (*RBL2)*, and cyclin-dependent kinase inhibitor 1B (*CDKN1B)*; the FOXO mediated cell cycle was identified as possible target for endotype-1 patients. Endotype-2 showed enriched inflammation-associated pathways, expressed by interleukin 6 (*IL6)*, matrix metallopeptidase *(MMP)1/3*, and vascular endothelial growth factor *(VEGF)*C and SASP pathways were identified as possible targets for endotype-2 patients. Notably, plasma-based metabolic profiles in overlapping blood samples (*N* = 21) showed two corresponding metabolic clusters in blood. These non-invasive metabolic profiles could function as biomarkers for patient-tailored targeting of senescence in OA.

**Supplementary Information:**

The online version contains supplementary material available at 10.1007/s11357-023-01001-2.

## Introduction

Osteoarthritis (OA) is a prevalent, age-related, and painful degenerative disease of joint tissues without curative treatment options [1]. Although all joint tissues have been implicated in OA, a hallmark of OA pathophysiology is the progressive degradation of articular cartilage. With the low reparative capacity of chondrocytes, the sole cell type in articular cartilage, important risk factors triggering OA pathophysiology are excessive mechanical stress and age [2, 3]. In this respect, we have previously studied downstream effects of hyper physiological mechanical stress applied to aged human ex vivo osteochondral explants and highlighted enrichment of GO-term “cellular senescence”. Senescence is one of the hallmarks of aging and is brought about by pro-survival strategies that cells use to avoid apoptosis upon cellular stress [3]. Nonetheless, such strategies in postmitotic cells, such as chondrocytes in articular cartilage, are likely different from the classical cellular senescence process where cells go into irreversible cell-cycle arrest, marked by permanent CDK inhibitor as p21^Cip1^ or p16^ink4a^ [4–6]. Therefore, senescence is more recently described as a complex process involving metabolic morphologic transformation of cells in response to cellular stressors, such as oxidative or mechanical stress [4, 7]. Notably, senescent cells express a harmful pro-inflammatory profile such as interleukin (*IL)1B*, *IL6*, matrix metalloproteinases (*MMP*) family member-*13*, and tumor necrosis factor A (*TNFA*), known as the senescence-associated secretory phenotype (SASP), that drives neighboring cells to also go into senescence [5, 8]. More specific, these SASP markers are well known to have an adverse effect on chondrocyte health, likely affecting propensity of chondrocytes to enter a disease OA-like state [6, 9]. Other lines of evidence imply the involvement of cellular senescence during OA pathophysiology such as the presence of senescence markers in OA tissue (e.g.p16^INK4A^ and p21) [4–6], age-related increase of senescence-associated beta galactosidase (SA-B-gal) activity, and increased presence of SASP markers in OA cartilage [6, 9, 10]. In mice, implantation of senescent cells into cartilage induced an OA-like environment, marked by the degeneration of cartilage and osteophyte formation [11].

The field has in recent years developed multiple drugs to target senescent cells either by selectively killing senescent cells (senolytics) or modifying their behavior (senomorphics) [12]. Due to the lack of disease modifying OA drugs [13] and the putative role of senescent cells in OA pathophysiology, these drugs have been suggested as a therapeutic option for OA [14]. Nonetheless, prior to such a therapeutic avenue, insight into the diversity of cellular senescence in aged articular cartilage is required [15]. Moreover, to allow translation of such knowledge into clinical practice, associated non-invasive biomarkers of the diversity must be developed. For that matter, metabolic features in the circulation are considered potent candidates, as they play a vital role in predicting vulnerability and frailty with aging [16–18].

Together, we here set out to characterize diversity of senescence in aged articular cartilage by analyzing a custom senescence-associated gene-set across our previously assessed mRNA-sequencing dataset [19]. Moreover, to allow translation to the clinic, we studied the potency of metabolic features in blood as corresponding biomarkers of this diversity in aged articular cartilage.

## Method

### Sample description

**Fig. 1:**
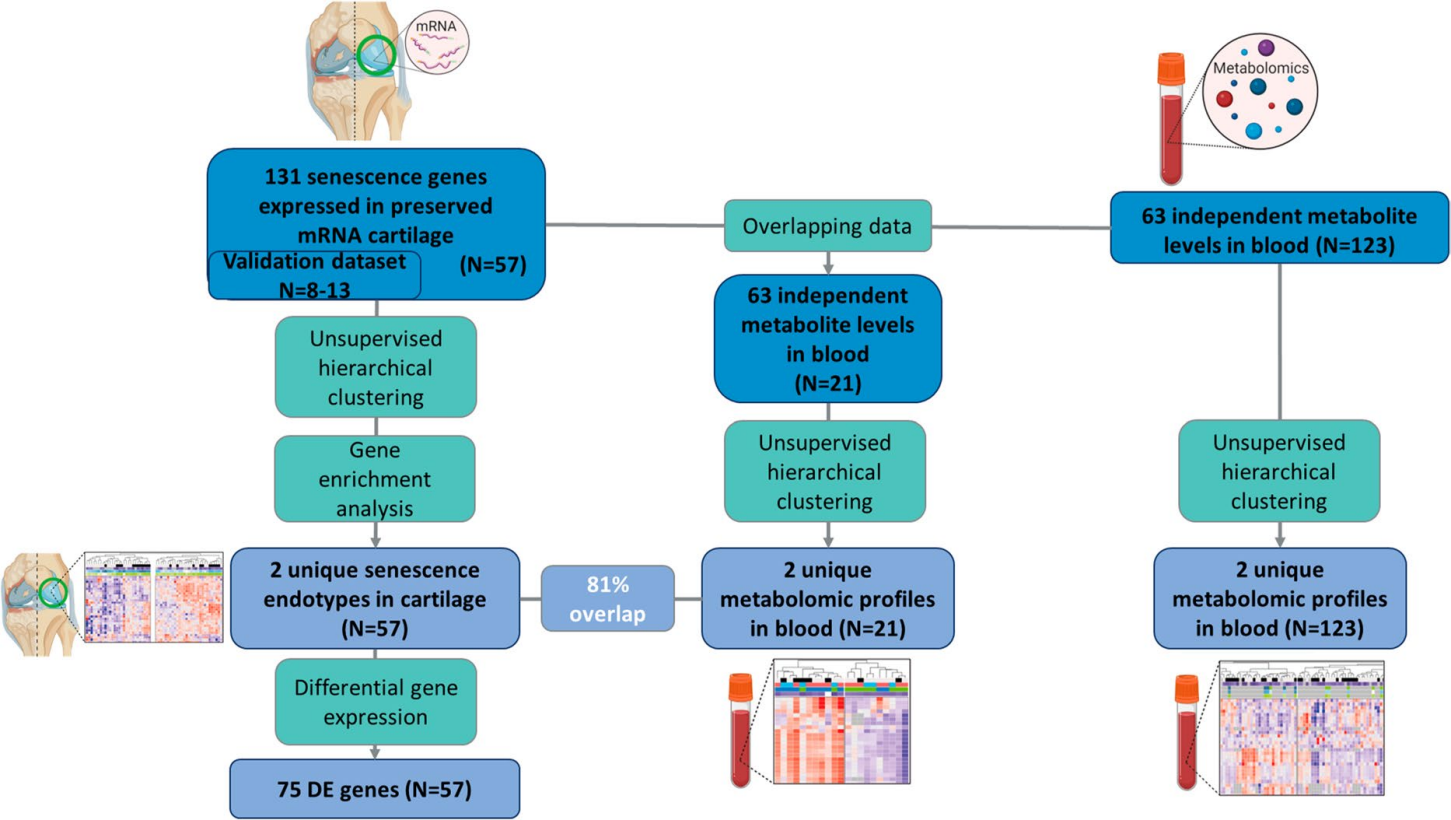
Study approach. 131 senescence genes were characterized in preserved cartilage (*N* = 57). Unsupervised hierarchical clustering and gene enrichment analysis were performed to find senescence endotypes in human aged cartilage. To find descriptive genes for the clusters, differential gene expression analysis was used. Metabolic blood profiles were extracted using unsupervised hierarchical clustering on metabolite levels in blood in an overlapping dataset with preserved cartilage (*N* = 21). Overlap between the cartilage endotypes and metabolic blood profiles was assessed. Unsupervised hierarchical clustering was performed on the total metabolite dataset (*N* = 123). Created with BioRender.com

Preserved (macroscopically healthy) cartilage (*N* = 57) as well as blood samples (*N* = 123) were obtained from patients who underwent joint replacement surgery due to end-stage OA (Research Arthritis and Articular Cartilage, RAAK study). In the current study, 57 preserved cartilage samples were used (Supplementary Table 1). From the 57 samples, there was an overlap of 21 patients of which preserved cartilage and blood data was available (Fig. 1). A senescence dataset was created for the current study, consisting of 155 senescence genes obtained from literature (Supplementary Table 2), from which 24 senescence genes were not expressed in OA cartilage (Supplementary Table 2). Therefore, further analysis was performed on 131 senescence genes expressed in cartilage.

### RNA sequencing

mRNA sequencing of the preserved and lesioned cartilage samples was previously achieved on the Illumina HiSeq 2000, HiSeq 4000, and HiSeq X ten. Quality control was performed using an in-house pipeline as described previously [7, 19]. In short, the data was corrected for batch effects and subsequently normalized and transformed using the variance stabilizing transform (VST) method.

### Unsupervised hierarchical clustering

To avoid looking at the end-stage OA phenotype, further analysis was performed on the 57 preserved cartilage samples and 131 expressed senescence genes. Unsupervised hierarchical clustering (ComplexHeatmap, v2.8.0 R package[20]) was performed on the normalized mRNA-seq data to identify different endotypes (clusters) of genes present in preserved cartilage (Fig. 1). Hierarchical clustering was performed on the expression levels of the 131 senescence genes, using Spearman’s correlation as distance and complete clustering as method. Expression levels of genes selected for endotypes-1 and -2 were validated in preserved cartilage tissue (*N* = 8–13, due to quality control, Fig. 3B) using quantitative reverse transcription PCR (RT-qPCR) adjusting for housekeeping genes POU class 6 homeobox 1 (*POU6F1*) and HECT domain E3 ubiquitin protein ligase 3 (*HECTD3*).

### Differential expression analysis

Differentially expression (DE) analysis was performed on the endotypes revealed with unsupervised hierarchical clustering present in preserved cartilage samples from the RAAK study [19]. DE analysis was performed as described previously [21]. In short, data was batch corrected and subsequently normalized and log2-transformed. *P*-values were Benjamini–Hochberg multiple testing corrected and < 0.05 was considered significant. Endotype-1 was used as baseline. All genes with a negative foldchange and FDR < 0.05 were retained to endotype-1 (Supplementary Table 3) and genes with a significant positive foldchange were retained to endotype-2 (Supplementary Table 4). A protein–protein interaction network was made using STRING v12.0 [22] with settings set to high confidence (0.700) and query proteins only.

### Pathway analysis

Pathway analysis was performed per endotype using the differentially expressed genes (Fig. 1). The pathway analysis was performed with DAVID v2021 [23] with a background correction for genes expressed in cartilage, selecting for GOTERM_BP_DIRECT, GOTERM_CC_DIRECT, GOTERM_MF_DIRECT, KEGG pathway, and REACTOME pathway. Subsequently, unique pathways (FDR significant, FDR < 0.05, and not expressed in the other endotype) were selected.

### Drug-gene interaction

Drug-gene interactions were found with the Drug Gene Interaction Database (DGIdb, v4.2.0 [24], filtering on approved drugs) using the 10 most significant differentially expressed genes for endotype-1 and -2. Further network analysis was performed using STITCH v5.0 [25] with high confidence (0.700).

### Metabolomic analysis

EDTA plasma samples (*N* = 123) were analyzed by Nightingale (Nightingale Ltd, Helsinki, Finland). This 1H-NMR technique resulted in 249 metabolic trait measurements. Quality control was performed; metabolic biomarkers with more than 5 below detection limit were removed from further analysis; and PCA analysis was performed to check for outliers (Supplementary Fig. 3). To limit the change of overfitting, 63 underived and independent metabolomic measurements were used for further analysis [17]. Data of 63 metabolic biomarkers and 123 patients was scaled using the scale function for the base package of R. The data of 21 patients (overlapping with cartilage mRNA, Fig. 1) was subsequently clustered using Spearman’s correlation distance and complete clustering using ComplexHeatmap v.2.8.0 R package [20]. Significant metabolic biomarkers between the metabolic endotypes were determined with a generalized equation estimate model using geese of geepack v.1.3.3 R package [26] with formula: Metabolic biomarker ~ Metabolic Endotype + Age + Sex + BMI. Subsequently, the data of 63 metabolic biomarkers and 123 patients was clustered using Spearman’s correlation distance and complete clustering using ComplexHeatmap v.2.8.0 R package.

## Results

### Aged human articular cartilage reveals a senescence signature

To investigate the role of senescence in aged articular cartilage, expression levels of 155 senescence-associated genes (Supplementary Table 2) were analyzed in a previously assessed RNA sequencing dataset of *N* = 57 macroscopically normal (preserved) articular cartilage of patients undergoing a joint replacement surgery due OA (The RAAK study, Supplementary Table 1) [19]. In silico exploration of the 155 senescence-associated genes showed 131 genes that had detectable expression levels (Supplementary Table 2). As shown in Supplementary Table 2, while the widely acknowledged senescent marker cyclin dependent kinase inhibitor (*CDKN*)*2A*, encoding p16INK4A, was lowly expressed in aged articular cartilage, *CDKN1A*, encoding p21, was highly expressed in aged articular cartilage. Among the highest expressed senescence-associated genes were fibronectin (*FN1)*, TIMP metallopeptidase inhibitor (*TIMP)1*, insulin-like growth factor binding protein *(IGFBP)5*, and *VEGFA*. On the other hand, among the factors marking the SASP, we recognized insulin-like growth factor (IGF) pathway; *IGFBP2* and *3*; and matrix metalloproteinases family members, *MMP3* and *MMP13* as being highly expressed (upper quartiles 3 and 4, Supplementary Table 2) in aged articular cartilage.

### Unsupervised hierarchical clustering of the senescence signature in aged human articular cartilage

**Fig. 2:**
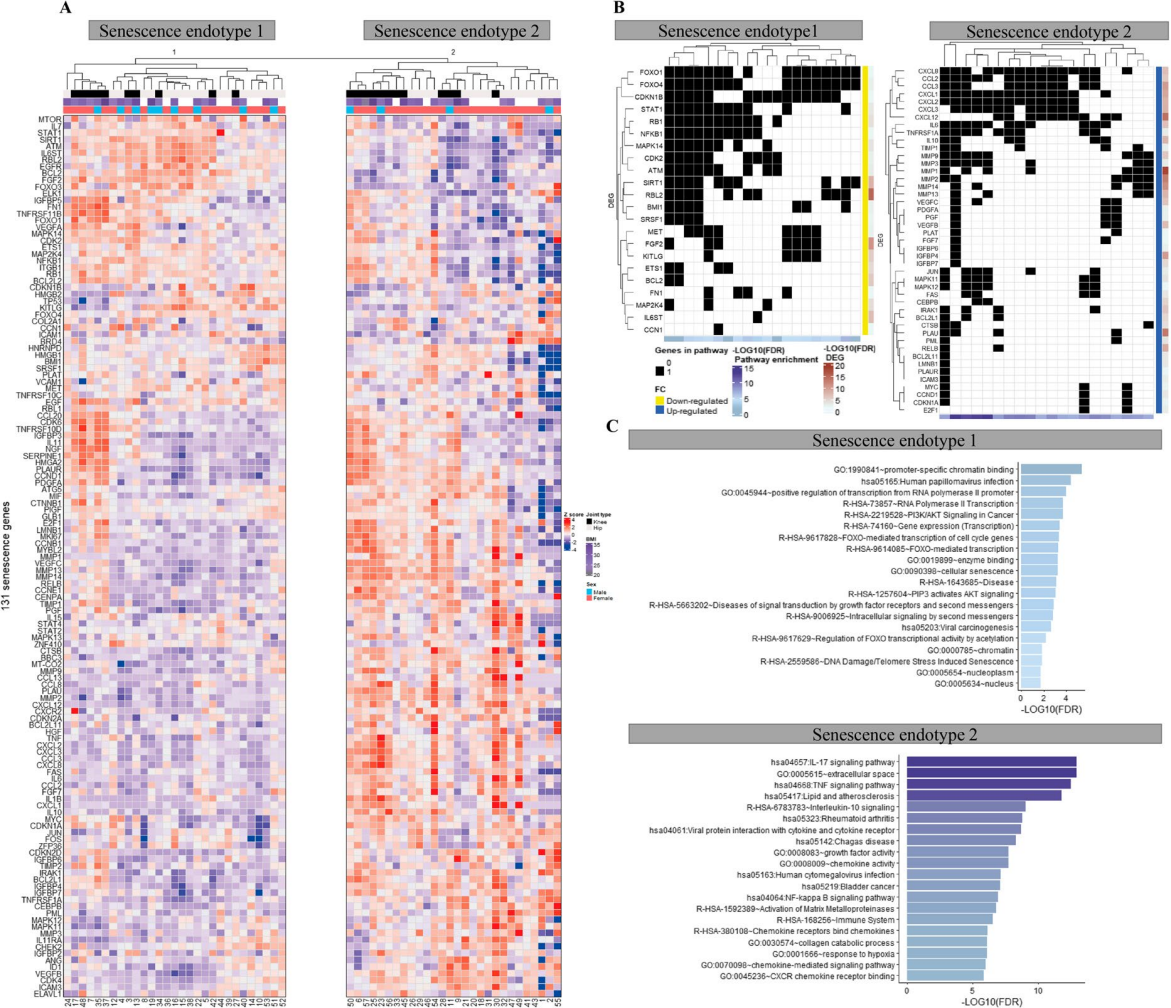
Unsupervised hierarchical clustering. **A** Heatmap of the two senescence endotypes. **B** Top 20 unique significant enriched pathways of the two senescence endotypes with their respective genes in black. **C** Barplot of the top 20 unique significant enriched pathways of the senescence endotypes. *N* = 57

To identify diversity of senescence processes in aged human articular cartilage, unsupervised hierarchical clustering was performed on RNA expression levels of the 131 senescence-associated genes (Fig. 1). Two distinct endotypes were identified, hereafter denoted as cartilage senescence endotype-1 and endotype-2 (Fig. 2A). To characterize the molecular senescent landscape between the endotypes further, differential gene expression analysis was performed. In total, 75 differential genes were identified (FDR < 0.05) with 24 genes marking endotype-1 related senescence (higher expressed in endotype-1, Supplementary Table 3) and 51 genes marking endotype-2 related senescence (higher expressed in endotype-2, Supplementary Table 4).

### Characteristics of senescence endotype-1 in aged articular cartilage

**Fig. 3:**
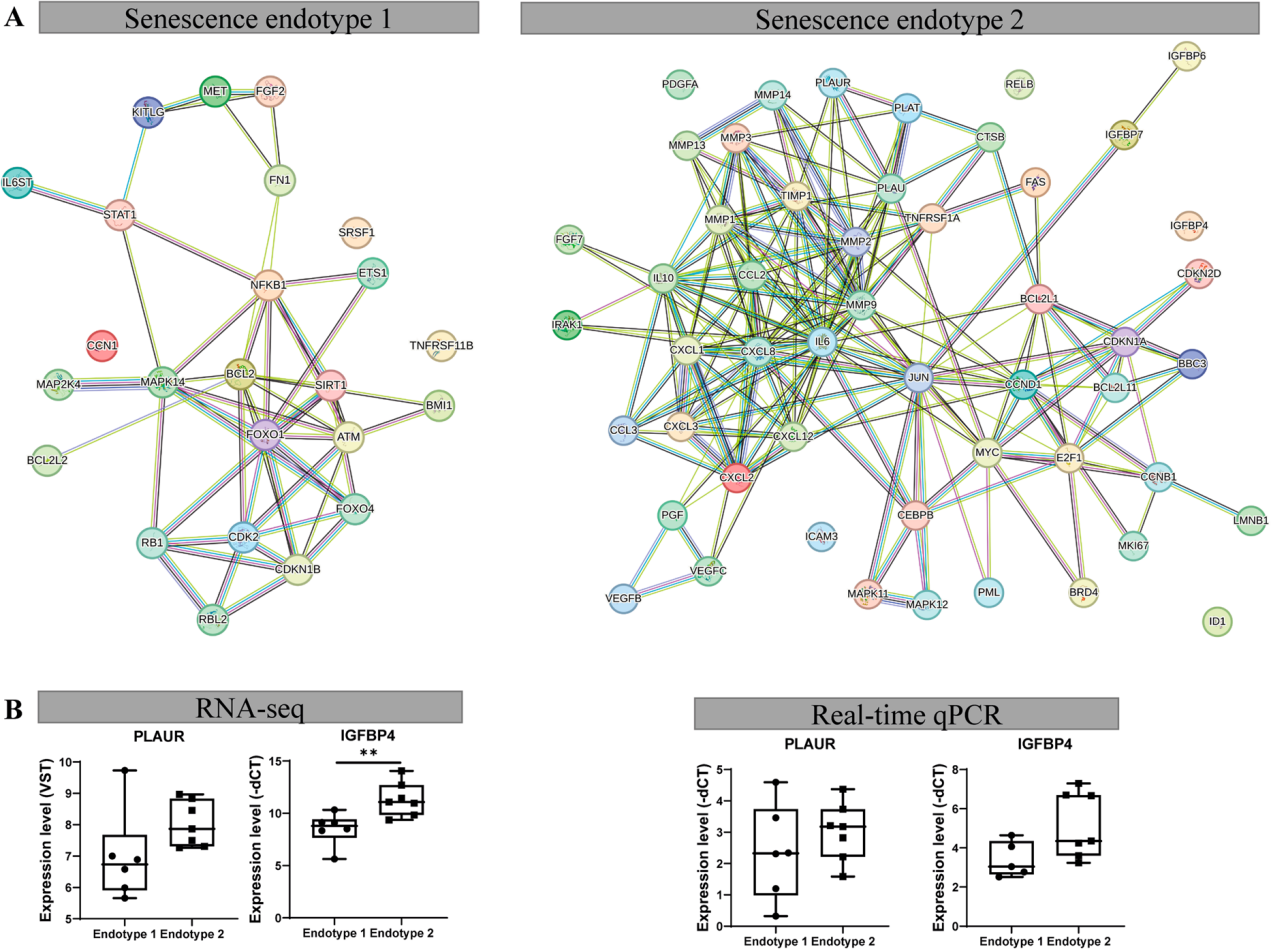
Characterization senescence endotypes. **A** STRING protein–protein network of the endotypes using endotype describing genes. **B** Technical validation of the genes expressed in the components. Measured by RNA-seq (left panel, *N* = 13) and validated by real-time qPCR (right panel, *N* = 8–13) showing senescence cartilage endotype-1 and -2. **P < 0.01, Student’s *t*-test

Figure 3A shows the protein–protein network of the identified endotype-1 related senescence genes with notable genes such as hub genes sirtuin 1 (*SIRT1)*, mitogen-activated protein kinase *(MAPK)14*, and cell proliferation regulators forkhead box O (*FOXO)1* and cyclin-dependent kinase (*CDK)2*. Next, to understand which unique biological processes play a role this component, we performed pathway analysis of the 24 genes marking senescence endotype-1 (Supplementary Table 3). To identify unique senescence pathways, overlapping processes between endotype-1 and -2 were excluded. As shown in Fig. 2B, C and supplementary Table 5, significant pathway enrichment was found for the 24 genes marking senescence endotype-1; cell proliferating pathways such as “FOXO-mediated transcription of cell cycle genes” (R-HSA-9617828 FDR = 4.4x10^−04^) with involvement of *FOXO1/4* and RB transcriptional corepressor like (*RBL)2*; “DNA damage/telomere stress-induced senescence” (R-HSA-2559586 FDR = 1.4x10^−02^) with involvement of *CDKN1B*, RB transcriptional corepressor *(RB)1*, and *CDK2*; and signaling pathways such as “intracellular signaling by second messengers” (R-HSA-9006925 FDR = 1.5x10^−03^) *RB1* and *CDKN1B*. In other words and as exemplified (Fig. 2A) and confirmed by RT-qPCR (Fig. 3B), patients that are expressed in endotype-1 showed higher expression of these genes compared to patients that express endotype-2 (Fig. 2A).

To gain further insight into possible treatments for endotype-1 patients, drug-gene interactions were investigated. When looking into previously known and approved drug-gene interactions, the 10 most significant differentially expressed genes of endotype-1, 70 drug-gene interactions were found (Supplementary Table 6). Network analysis of this interaction showed a highly interconnected network, including many drug-drug interactions (Supplementary Fig. 1). Worthy notions are metformin, rapamycin, and dasatinib, which all showed direct connections to the top 10 genes. Metformin showed direct connections to TNF receptor superfamily member (*TNFRSF)11B*, ATM serine/threonine kinase *(ATM)*, and *SIRT1*; rapamycin to MET proto-oncogene (*MET)*, *CDK2*, *RB1*, *ATM*; and dasatinib to *MET* and *MAPK14* (Supplementary Fig. 1). This indicates the need for further analysis to select possible treatable targets.

### Characteristics of senescence endotype-2 in aged articular cartilage

Figure 3A shows the protein–protein network of the identified endotype-2 related senescence genes with, among others notable SASP genes such as *MMP1/13*, plasminogen activator, urokinase receptor (*PLAUR)*, chemokines C-X-C motif chemokine ligand* (CXCL)1–3*, *IL6*, and *IGFBP4/6/7*. As shown in Fig. 2B, C and supplementary Table 7, pathway enrichment analysis of the 51 descriptive endotype-2 genes showed enrichment for inflammatory and signaling pathways, such as “IL17 signaling pathway” (hsa04657 FDR = 1.0x10^−13^, Fig. 2B, C) and “TNF signaling pathway” (hsa04668 FDR = 2.8x10^−13^, Fig. 2B, C) represented by genes such as *IL6*, *CXCL1-3*, *MMP3/9/14*, and *VEGFC*, respectively. As well as inflammatory related diseases such as “Rheumatoid arthritis” (FDR = 1.6x10^−09^) with *MMP1/3* and *IL6*.

Analysis of drug-gene interactions of endotype-2 genes revealed 49 drug-gene interactions for the 10 most significantly differentially expressed genes in endotype-2 (supplementary Table 8). Further network analysis showed for endotype-2 highly interconnected drug-gene and drug-drug interactions (Supplementary Fig. 2). Notable drugs are doxycycline, zoledronic acid, alendronate, and calcitriol; these drugs show a direct connection to the top 10 genes of endotype-2 and are located closely together in the network (Supplementary Fig. 2). Doxycycline showed direct connection to *MMP2* and *MMP14*; zoledronic acid and alendronate to *MMP2*; and calcitriol to *IGFBP4*, RELB proto-oncogene, NF-KB subunit *(RELB)*, *MMP14*, *CXCL12*, and *MMP2*. Alendronate also showed direct connections to zoledronic acid and calcitriol.

Taken together, we could successfully stratify patients for 2 different senescent endotypes in human aged cartilage, endotype-1, marked by changes in cell proliferating pathways/absence of inflammation and increased expression of *FOXO1/4*, *RBL2*, and *CDKN1B*, and endotype-2, marked by inflammation/cell cycle regulating pathways and upregulated gene expression of *IL6*, *MMP1/3*, and *VEGFC*. We have indicated possible drugs that target endotype-specific genes. These findings indicate different intrinsic expected survival pathways of patients. OA is closely related to age and is the result of age-related changes. This raises the question whether the senescence endotypes reflect a body-wide senescence state rather than a tissue-specific profile.

### Metabolomic profiles in blood reflect a body-wide senescence state

Since metabolic biomarkers are excellent aging biomarkers, we next set out to measure metabolic biomarkers using the 1H-NMR (Nightingale) platform and explored whether the tissue-specific senescent endotypes identified in articular cartilage correspond to any profile in the circulation. Hereto, we measured metabolic biomarkers in 123 serum samples that had an overlap of 21 individuals for which we analyzed the RNA sequencing data of articular cartilage (The RAAK study). Metabolic biomarkers were excluded below a detection limit of 5 and PCA analysis was used to identify outliers (Supplementary Fig. 3). To limit the chance of overfitting, 63 underived and independent metabolomic biomarkers were used for further analysis [17]. These 63 metabolic biomarkers compose of lipids, fatty acids, amino acids, and glycolysis-related metabolites.

**Fig. 4:**
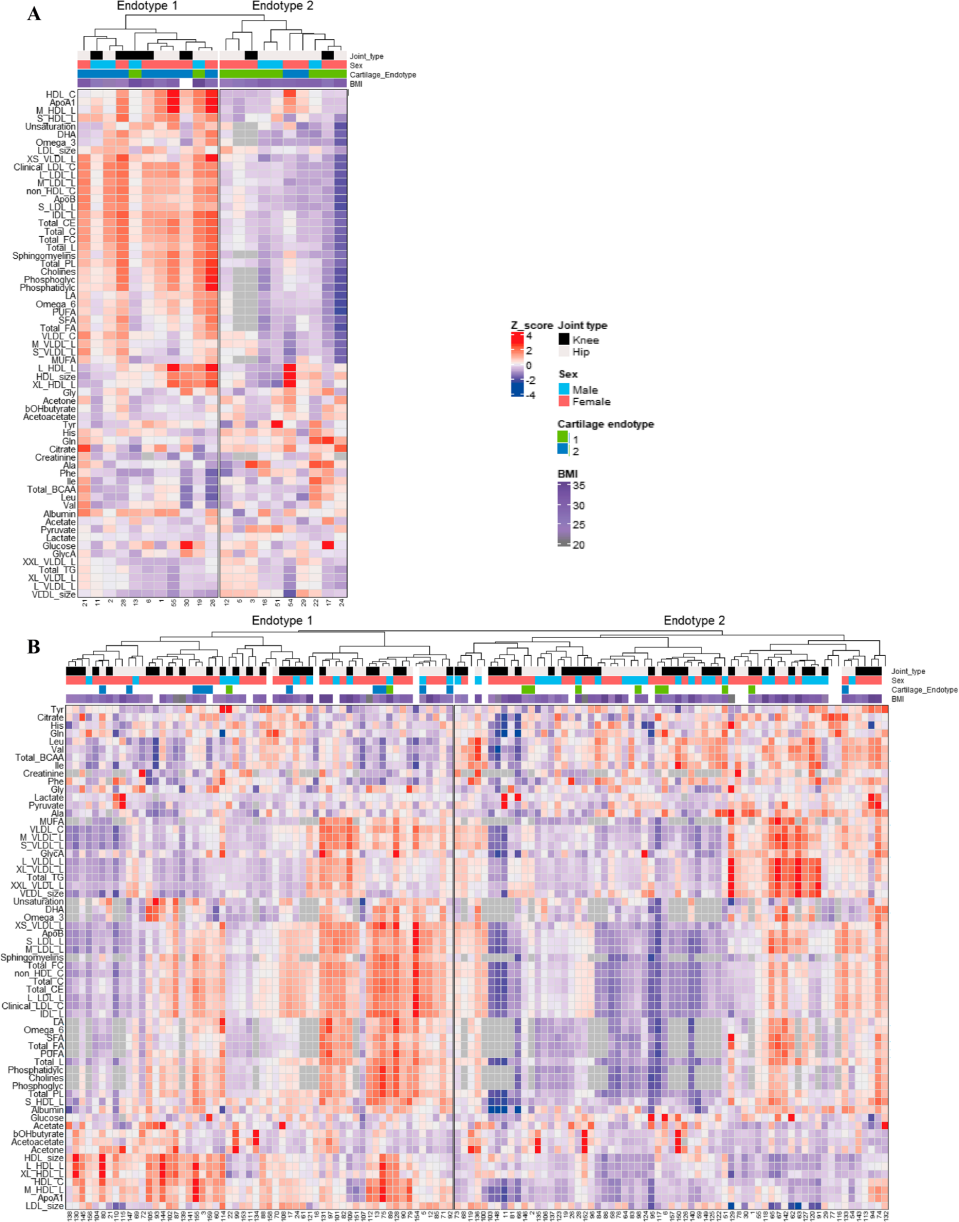
Serum metabolite measurements. Clustering of serum metabolite expression levels (*Z*-score) based on Spearman’s correlation resulted in two similar clusters as on cartilage mRNA level. **A** In the overlapping dataset, 81% was correctly clustered (*N* = 21). **B** In all metabolite samples, 86% was correctly clustered (*N* = 123)

Unsupervised hierarchical clustering of the 63 independent metabolic biomarkers in the 21 overlapping patients resulted in two distinct metabolic clusters (Figs. 1 and 4A, Supplementary Table 9) independent of BMI (FDR = 4.2x10^−1^, Supplementary Table 10A). These two metabolic clusters corresponded for 81% to the articular cartilage endotypes, where metabolic endotype-1 corresponds to cartilage endotype-2 and metabolic endotype-2 with cartilage endotype-1 (Figs. 2A and 4A). Herein, the two endotypes appear to have opposite profiles with respect to lipids, apolipoproteins, and fatty acids (Fig. 4A and supplementary Table 11). Senescence endotype-1 was marked by amino acids: histidine (FDR = 1.7x10^−2^, Supplementary Table 11) and phenylalanine (FDR = 2.3x10^−2^), while senescence endotype-2 was marked by cholesterol (FDR = 1.5x10^−20^, Supplementary Table 11), total lipids in low density lipids (with a diameter variating from 18.7 to 25.5 nm, FDR = 1.2x10^−27^), apolipoproteins A1 and B (FDR = 6.2x10^−4^ and FDR = 3.0x0^−19^, respectively), fatty acids (FDR = 9.86x10^−5^) mainly by poly unsaturated fatty acids (PUFA, FDR = 3.3x10^−5^), and omega 3 and 6 (FDR = 7.6x10^−6^ and FDR = 1.5x10^−4^, respectively). Moreover, including all 123 patients with metabolic data confirmed that these metabolic clusters were robust (Fig. 4B) independent of BMI (FDR = 3.3x0^−1^, Supplementary Table 10B).

## Discussion

In the present study, senescent heterogeneity in aged articular cartilage was characterized and associated with circulating metabolic biomarkers. Based on the RNA sequencing profile of 131 expressed senescence genes, two robust cellular senescent endotypes were identified in aged articular cartilage. Notable was that unsupervised hierarchical clustering of 63 independent metabolic biomarkers resulted in two clusters that had a significant large overlap (81%) with the identified senescence endotypes in cartilage. Senescence endotype-1 was characterized in aged articular cartilage by genes involved in cell proliferation such as *FOXO4* and *CDKN1B* and concomitantly in plasma with amino acids such as histidine. Senescence endotype-2, on the other hand, was characterized by genes involved in SASP such as *IL6* and *MMP1/3* and alongside in serum with (low density) lipids, cholesterol, and fatty acids. Finally, we explored drug-gene interactions of the endotype-specific pathways that, together with the metabolic biomarkers, could facilitate patient-tailored targeting of senescence in OA.

The identified senescence endotype-1 in aged articular cartilage was characterized by FOXO-mediated transcription of cell cycle genes and particularly *FOXO4* and *CDKN1B* (encoding p27). These FOXO mediated proteins control several functions such as cell survival, growth, and metabolism, likely reflecting the classic cell cycle arrest feature of senescence via p21 [29, 30]. Herein, FOXO4 can directly bind to p53 when DNA damage occurs, activating p53-dependent transcription of p21 [30]. P27, part of the CDK inhibitor family, is involved in the cell cycle progression and responsive to extracellular antiproliferative signals. Increased levels of p27 resulted in reduced levels of proliferation and cytoplasmic p27 regulates the actin skeleton, affecting cell migration [31]. The therapeutics identified for endotype-1 pathways, metformin, rapamycin, and dasatinib, all affect FOXO-mediated transcription of cell cycle [32–35], where metformin regulates FOXO proteins by regulating mechanistic target of rapamycin kinase (mTOR) signaling through 5′adenosine monophosphate-activated protein kinase (AMPK) activation, which in turn activates FOXO proteins [32] [33]. Dasatinib inhibits phosphorylation of FOXO proteins, resulting in apoptosis and targets the phosphatidylinositol 3′-kinase- protein kinase B (PI3K-AKT) signaling pathway, which in its turn inhibits FOXO activity [34, 35]. This is in line with the increased blood metabolite levels of amino acids, able to regulate cell cycle arrest [36], of senescence endotype-1 patients. Taken together, the presence of important endotype-1 describing genes in FOXO-mediated cell cycle regulation, (in)direct link of the drugs to the FOXO cell cycle regulation, and increased levels of amino acids in the metabolic blood profile suggest that endotype-1 patients could benefit from treatments targeting the FOXO-mediated transcription of cell cycle pathway.

The identified senescence endotype-2 in aged articular cartilage was characterized inflammatory SASP pathways and particularly *IL6* and *MMP1/3*. IL6 is a main pro-inflammatory cytokine, MMP1, and 3 members of the MMP family and can control the factors present in the SASP by cleaving several soluble factors [37]. These MMPs are also present in the OA pathophysiology, cleaving main components of the cartilage matrix, collagen type 2, and aggrecan. The therapeutics identified for endotype-2 pathways, doxycycline, zoledronic acid, alendronate, and calcitriol, all affect the inflammatory SASP [38–41], where doxycycline is a strong anti-inflammatory drug that can inhibit MMP activity as well as modulate cell proliferation and reduce the SASP release [38, 39], while zoledronic acid decreases the amount of senescent cells and dampens the SASP profile in vitro in human lung fibroblasts as well as in vivo in aged mice [40]. This is in line with the inflammatory metabolic health of endotype-2 patients, marked by the increase of lipids, cholesterol, and fatty acids [42, 43]. The presence of important SASP genes, the anti-inflammatory effects of the drugs, and presence of an inflammatory metabolic profile suggest that endotype-2 patients could benefit most from therapeutics targeting inflammatory SASP pathways and not only the cell cycle arrest pathways.

Prior to any clinical application it should, however, be taken into account that the proposed pharmacological treatments are primarily put forward based on theoretical drug-gene interactions. Hence, comprehensive assessment, encompassing both the strength and weaknesses of the proposed pharmacological treatments, is required as well as additional pre-clinical testing of drugs. We propose that such pre-clinical studies for applications to treat OA should preferably be done in aged human osteochondral explants challenged by hyper-physiological stress [7].

The presence of two robust senescence endotypes in aged articular cartilage highlights heterogeneity in the response of chondrocytes upon environmental stressors throughout life. Particularly remarkable is that these cartilage-specific endotypes were robustly reflected by two corresponding metabolomic profiles in the circulation, independent of BMI. Although these blood metabolic profiles could be directed entirely by the senescence endotype in articular cartilage, we feel that it is more likely the reflection of intrinsic, subject-specific, survival strategy performed in all tissues. This is particularly because chondrocytes are metabolically silent and reside in a non-perfused tissue [27]. Moreover, Farr and colleagues [28] recently demonstrated that systemic administration of senolytics, hence body-wide clearance, was more effective to reduce bone dysfunction as compared to local administration in mice. This support our notion that tissue-specific senescent endotypes actually reflect intrinsic, subject-specific, survival strategy performed in all tissues, whereas the metabolic profiles could facilitate patient-tailored treatment options for OA. In any case, more studies are necessary to confirm these hypotheses, preferably by assessing senescence endotypes in other tissues that are subjected to age-related degenerative changes (e.g., muscle) and in comparison to metabolic profiles in the blood. Moreover, despite the fact that the two senescence endotypes were identified by unsupervised hierarchical clustering in two different molecular levels of information (transcriptomics and metabolomics), the robustness and generalizability of the two identified senescence endotypes need to be further validated in independent cohorts. On a different note, if metabolic profiles indeed reflect intrinsic conditions in the body, they could also originate from associated co-morbidities such as metabolic diseases or cardiovascular diseases. Consequently, the suggested pharmacological therapies may not be exclusive, hence requiring caution and pre-clinical testing prior to clinical applicability.

Taken together, our study showed two articular cartilage-specific senescence endotypes with corresponding metabolic profiles in blood. Given that articular cartilage is a metabolically silent and non-perfused tissue, we advocate that the metabolic profiles in blood could be a reflection of an intrinsic, body-wide, survival strategy of tissues. Consequently, these non-invasive metabolic profiles could function, as biomarkers for patient-tailored targeting of senescence in OA and beyond.

### Supplementary Information

Below is the link to the electronic supplementary material.

Supplementary file1 (DOCX 8476 KB)

Supplementary file2 (XLSX 54.3 KB)

## Data Availability

The mRNA sequencing data of the articular cartilage is deposited at ArrayExpress (E-MTAB-7313). Data used in this study are available upon reasonable request sent to the corresponding author.
