# Supplementary material for: Identified senescence endotypes in aged cartilage are reflected in the blood metabolome
Source: GeroScience. 2023 Nov 14;46(2):2359–69. doi: 10.1007/s11357-023-01001-2 (PMC10828277; doi:10.1007/s11357-023-01001-2)
Supplement: Supplementary file 1 — Supplementary file1 (DOCX 8476 KB) [file 11357_2023_1001_MOESM1_ESM.docx]

Identified senescence endotypes in aged cartilage are reflected in the blood metabolome

I. Boone^1^, M. Tuerlings^1^, R. Coutinho De Almeida^1^, J. Lehmann^2^, Y.F.M. Ramos^1^, R.G.H.H. Nelissen^3^, P.E. Slagboom^1,4^, P.L.J. Keizer^2,5^, I. Meulenbelt^1^

1 Dept. of Biomedical Data sciences, section Molecular Epidemiology, Leiden University Medical Center, Leiden, The Netherlands

2 Center for Molecular Medicine, Division of Laboratories, Pharmacy and Biomedical Genetics, University Medical Center Utrecht, Utrecht, The Netherlands

3 Dept. of Orthopaedics, Leiden University Medical Center, Leiden, The Netherlands

4 Max Planck Institute for Biology of Aging Cologne Germany

5 Cleara Biotech B.V. Utrecht, The Netherlands

**Corresponding author:**

Ingrid Meulenbelt, PhD,

Section of Molecular Epidemiology,

Department of Biomedical Data Sciences,

Leiden University Medical Center,

LUMC Post- zone S- 05- P, PO Box 9600, 2300 RC Leiden, The Netherlands.

Email: [i.meulenbelt@lumc.nl](mailto:i.meulenbelt@lumc.nl).

Supplementary figures

**Fig S1** Drug-gene interactions of the 10 most significant differentially expressed genes for endotype 1. Green edges represent drug/gene interactions and red edges represent drug/drug interactions. Edges represent protein-protein associations, associations are meant to be specific and meaningful, i.e. proteins jointly contribute to a shared function; this does not necessarily mean they are physically binding each other. Edge Confidence:
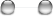
low (0.150)
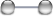
high (0.700)
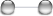
medium (0.400)
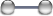
highest (0.900)

**Fig S2** Drug-gene interactions of the 10 most significant differentially expressed genes for endotype 2. Green edges represent drug/gene interactions and red edges represent drug/drug interactions. Edges represent protein-protein associations, associations are meant to be specific and meaningful, i.e. proteins jointly contribute to a shared function; this does not necessarily mean they are physically binding each other. Edge Confidence:
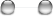
low (0.150)
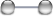
high (0.700)
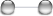
medium (0.400)
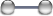
highest (0.900)


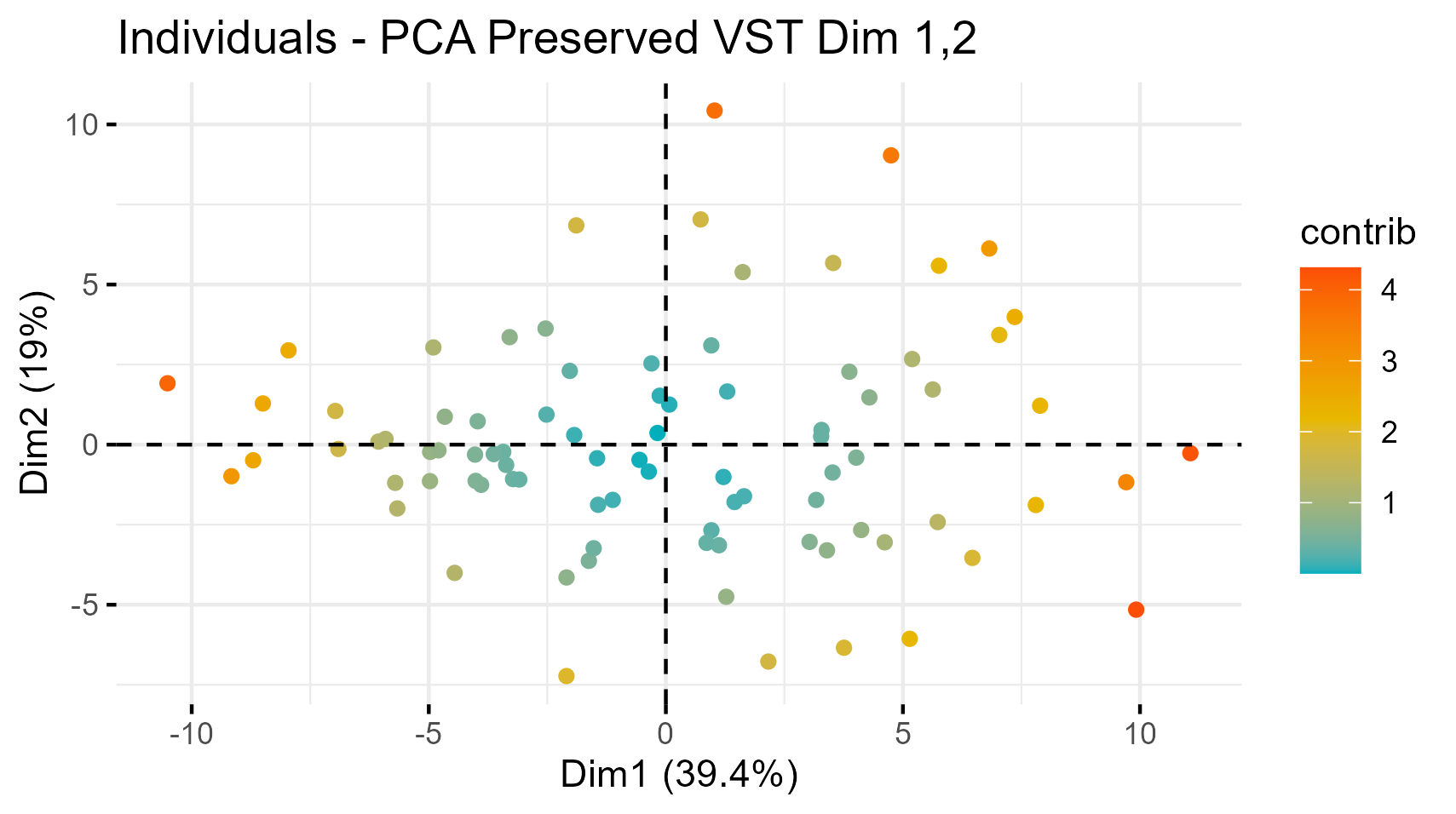


**Fig S3** PCA plot of component 1-2. No sample outliers were detected.
